# Uterine fibroid embolisation: advocating women’s choice for uterine salvage

**DOI:** 10.1186/s42155-020-00161-y

**Published:** 2020-10-20

**Authors:** Warren Clements

**Affiliations:** 1 Department of Radiology, Alfred Health, Alfred Hospital, 55 Commercial Road, Melbourne, VIC 3004 Australia; 2 Department of Surgery, Monash University, Melbourne, Australia; 3 National Trauma Research institute, Monash University, Melbourne, Australia

To the Editor,

I read with interest the recent articles by de Bruijn et al. (2019) and Makris et al. (2020). Both articles articulate well the current disparity between the number of uterine fibroid embolisation (UFE) procedures being performed each year compared with the number of hysterectomies in their respective countries.

As eluded to by Makris et al. (although without reference), a disparity also exists in Australia. My group recently showed that Australian Medicare data paints a similar picture. Extrapolating the initial data to the last 7 years, there has been an average of 163 Medicare-funded UFE procedures each year compared with an average of 30,757 uterine surgeries of which 13,126 were for hysterectomy (Clements et al. 2020a; Yusuf et al. 2016). It is estimated that 20% of uterine surgeries in Australia are for fibroid disease (Australian Government Department of Human Services 2020) which generates an estimated average of 6124 surgeries for fibroid disease per year (Fig. 1). UFE makes up a relative percentage of only 2.7% of fibroid procedures compared with surgery.

**Fig. 1:**
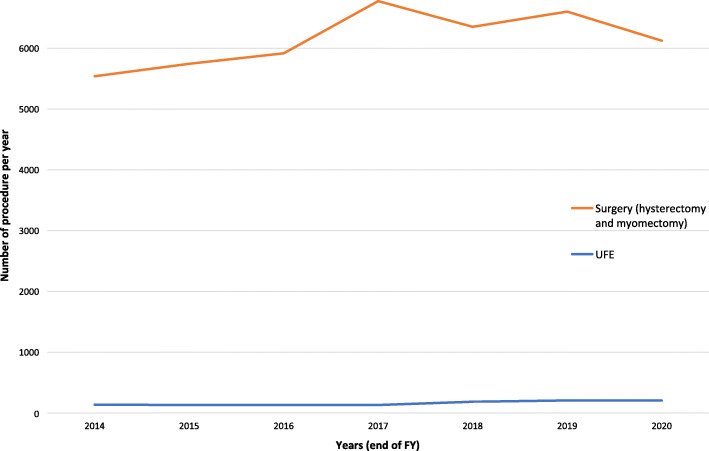
Graph comparing the estimated number of uterine surgeries for fibroid disease, compared to the number of uterine fibroid embolisation procedures, each financial year in Australia. Data is extrapolated from Medicare item codes 35,410, 35,649, 35,638, 35,653, 35,661, 35,657, 35,753, 35,754, and 35,756, for the financial years 2013/14 to 2019/20

No doubt, one of the major reasons for this disparity is that treatment referrals usually come from gynaecologists and a degree of scepticism remains around the efficacy of UFE amongst this group. However, the recent FEMME trial supports previous studies including EMMY and REST, showing that UFE is a safe and efficacious procedure (Manyonda et al. 2020). While the FEMME trial showed better quality of life scores for myomectomy than for UFE, this was offset by higher complications (particularly haemorrhage requiring transfusion), longer hospital stay, and more patients in the UFE group reported a pregnancy. Coupling this with the low cost to perform (Clements et al. 2020b), the data to support UFE has never been more robust, and we must now permanently put scepticism in the graveyard.

As pointed out by Makris et al., better advocacy of the procedure including increasing public awareness of Interventional Radiology as a specialty is beyond overdue, and will lead to streamlined referral pathways and better multidisciplinary collaboration. However, we must consider that the ultimate goal we are looking to achieve is that of patient advocacy – allowing women to have the right to choose the best and individualised treatment for them. For many women, the long-term psychological aspect of hysterectomy is paramount, and must not be dismissed (Khan et al. 2020). Hysterectomy also has a higher association with cardiovascular disease and mortality (Varol et al. 2001).

We must not sit back, but rather all interventional radiologists have a mandate to provide advocacy for patients and continue to build evidence to support our craft. The ultimate goal is to allow all women to have access to consultation with an interventional radiologist, and thus to make an informed evidence-based decision that is right for them.

## Data Availability

The datasets used and/or analysed during the current study are available from the corresponding author on reasonable request.

## Abbreviation

UFE: Uterine fibroid embolisation
